# From Vineyard to Vision: Efficacy of Maltodextrinated Grape Pomace Extract (MaGPE) Nutraceutical Formulation in Patients with Diabetic Retinopathy

**DOI:** 10.3390/nu16172850

**Published:** 2024-08-26

**Authors:** Elisabetta Schiano, Sabrina Vaccaro, Vincenzo Scorcia, Adriano Carnevali, Massimiliano Borselli, Domenico Chisari, Fabrizia Guerra, Fortuna Iannuzzo, Gian Carlo Tenore, Giuseppe Giannaccare, Ettore Novellino

**Affiliations:** 1 Inventia Biotech-Healthcare Food Research Center s.r.l., Strada Statale Sannitica KM 20.700, 81020 Caserta, Italy; elisabettaschiano@inventiabiotech.com (E.S.); fabrizia.guerra@inventiabiotech.com (F.G.); ettorenovellino@inventiabiotech.com (E.N.); 2 Department of Ophthalmology, University Magna Graecia of Catanzaro, 88100 Catanzaro, Italy; sabrina_vaccaro@libero.it (S.V.); vscorcia@unicz.it (V.S.); adrianocarnevali@unicz.it (A.C.); mborselli93@gmail.com (M.B.); domenico.chisari@studenti.unicz.it (D.C.); 3 Department of Pharmacy, University of Chieti-Pescara G. D’Annunzio, 66100 Chieti, Italy; fortuna.iannuzzo@unich.it; 4 Department of Pharmacy, University of Naples Federico II, Via Domenico Montesano 59, 80131 Naples, Italy; giancarlo.tenore@unina.it; 5 Eye Clinic, Department of Surgical Sciences, University of Cagliari, 09124 Cagliari, Italy

**Keywords:** diabetic retinopathy, grape pomace polyphenols, nutraceuticals, diabetes, macular degeneration, oxidative stress

## Abstract

Despite recent advances, pharmacological treatments of diabetic retinopathy (DR) do not directly address the underlying oxidative stress. This study evaluates the efficacy of a nutraceutical formulation based on maltodextrinated grape pomace extract (MaGPE), an oxidative stress inhibitor, in managing DR. A 6-month, randomized, placebo-controlled clinical trial involving 99 patients with mild to moderate non-proliferative DR was conducted. The MaGPE group showed improvement in best-corrected visual acuity (BCVA) values at T3 (*p* < 0.001) and T6 (*p* < 0.01), a reduction in CRT (at T3 and T6, both *p* < 0.0001) and a stabilization of vascular perfusion percentage, with slight increases at T3 and T6 (+3.0% and +2.7% at T3 and T6, respectively, compared to baseline). Additionally, the levels of reactive oxygen metabolite derivatives (dROMs) decreased from 1100.6 ± 430.1 UCARR at T0 to 974.8 ± 390.2 UCARR at T3 and further to 930.6 ± 310.3 UCARR at T6 (*p* < 0.05 vs. T0). Similarly, oxidized low-density lipoprotein (oxLDL) levels decreased from 953.9 ± 212.4 µEq/L at T0 to 867.0 ± 209.5 µEq/L at T3 and markedly to 735.0 ± 213.7 µEq/L at T6 (*p* < 0.0001 vs. T0). These findings suggest that MaGPE supplementation effectively reduces retinal swelling and oxidative stress, contributing to improved visual outcomes in DR patients.

## 1. Introduction

Diabetic retinopathy (DR) is one of the most common complications of diabetes mellitus (DM), affecting approximately 22.27% of the diabetic population [1]. It represents the leading cause of preventable blindness among the working-age population, thereby posing a significant public health concern [2]. Future projections suggest a notable escalation in the global prevalence of DR, with the affected population anticipated to rise from 103 million in 2020 to 160 million by the year 2045. This increase is concomitant with the forecasted expansion of the global diabetic population, which is estimated to grow from 463 million in 2019 to 700 million in 20 years [3]. The pathogenesis of DR is complex, encompassing multiple mechanisms such as the accumulation of Advanced Glycation End Products (AGEs), oxidative stress, sorbitol accumulation, activation of protein kinase C, upregulation of the renin–angiotensin system, and vascular endothelial growth factor (VEGF) [4,5,6]. Elevated levels of VEGF, along with other pro-inflammatory mediators, compromise the integrity of the blood–retinal barrier, leading to increased vascular permeability and reduced fluid clearance, culminating in the onset of macular edema [7,8]. Diabetic macular edema (DME), the main cause of vision loss in diabetic individuals, is characterized by a gradual increase in retinal thickening, ultimately compromising the central macular region and adversely affecting visual acuity [9]. Therefore, among the key ocular manifestations of diabetic retinopathy, alterations in best-corrected visual acuity (BCVA) and central retinal thickness (CRT) stand out as critical indicators of DR progression and treatment efficacy [10].

The management of DR poses a substantial clinical challenge, requiring novel approaches for both prevention and intervention. The occurrence of spontaneous resolution of DME is quite rare. More often, regression of DME is observed when systemic risk factors, such as glycemic regulation, blood pressure management, or lipid control, are modified [11]. Additionally, the heterogeneity of disease presentation and response to treatment underscores the complexity of DR management [12]. The current literature emphasizes the efficacy of intravitreal injections of anti-VEGF agents and/or corticosteroids in managing DME in most cases. [13]. However, complete resolution of edema remains difficult to achieve in a significant proportion of patients, or the degree of retinal thinning might not justify intravitreal injection therapy [14].

Despite recent advances, pharmacological treatments primarily target specific pathways implicated in the disease’s pathogenesis, such as VEGF inhibition, rather than oxidative stress. Oxidative stress is recognized as a pivotal factor in DR, influencing the regulation of various biochemical pathways, mitochondrial dysfunction, and hypoxia-driven VEGF synthesis [15]. Therefore, the investigation into oxidative stress as a central mechanism in the pathogenesis of DR has prompted significant interest in exploring the efficacy of inhibitors of oxidative damage [4]. These interventions may include the use of antioxidant compounds. These substances are able to restore the redox balance within the retina, enhance endogenous antioxidant defenses or modulate oxidative stress-related signaling pathways [16].

In this scenario, nutraceuticals offer a promising avenue for adjunctive therapy due to their pleiotropic effects, including anti-inflammatory, antioxidant, and neuroprotective properties. Notably, orally administered polyphenols have demonstrated the ability to cross the blood–retina barrier, further highlighting their potential utility in treating retinal disorders [17,18]. This has prompted several randomized clinical trials to evaluate the efficacy of various bioactive molecules, including polyphenols, in the management of retinal degenerative diseases. Among the various sources of nutraceuticals, grape pomace, a byproduct of the winemaking process, has gained particular attention for its high content of bioactive constituents. This waste product contains a wide range of bioactive molecules, including polyphenols, flavonoids and anthocyanins, all recognized for their positive effects on ocular health due to their numerous biological activities, including inhibition of oxidative stress, modulation of inflammatory responses and promotion of vascular integrity [19,20]. Both preclinical and clinical studies have provided evidence supporting the potential of grape pomace-derived nutraceuticals in mitigating the progression of DR and preserving visual function. In this regard, an in vivo study conducted by Sun et al. demonstrated the protective effect of grape seed proanthocyanidin extract (GSPE) on the retina by ameliorating oxidative stress-mediated injury through activation of the nuclear erythroid 2-related factor 2 (Nrf2) pathway [21]. Furthermore, Wan and colleagues reported significant reductions in retinal pigment epithelium (RPE) cellular senescence following GSPE supplementation in both in vitro and in vivo age-related macular degeneration (AMD) models, suggesting a potential therapeutic role on degenerative retinal dysfunction [22]. Nonetheless, a very recent study investigated the beneficial potential of 6-month supplementation with grape pomace extract (GPE) on AMD [23]. The study revealed a significant reduction in CRT and a concomitant improvement in visual acuity, emphasizing the promising role of maltodextrinated grape pomace extract (MaGPE). Moreover, the treatment was associated with a marked decrease in serum concentrations of trimethylamine-N-oxide (TMAO) and circulating levels of oxidative stress biomarkers, highlighting the antioxidant and vascular-protective properties of grape pomace polyphenols as the underlying mechanisms. This preliminary exploration, part of a pilot clinical trial, suggests the potential of this byproduct matrix as a useful strategy for eye disease management, particularly through the attenuation of oxidative stress and vascular dysfunctions associated with the progression of the disease.

Starting from this evidence, the present study aims at investigating the efficacy of a nutraceutical formulation based on MaGPE in the management of DR by evaluating changes in ophthalmic parameters, mainly BCVA and CRT.

## 2. Materials and Methods

### 2.1. Study Population and Design

This study was a monocentric, randomized, placebo-controlled clinical trial involving patients with DR who were recruited at the Department of Ophthalmology, University Magna Græcia (Catanzaro, Italy) from December 2020 to September 2022. The study was approved by the local ethics committee (Calabria Region Ethics Committee, Central Area Section, approval n. 311, date 17 September 2020). This study is listed on the ISRCTN registry (www.isrctn.com, accessed on 8 July 2024) with ID ISRCTN15020073 (https://www.isrctn.com/ISRCTN15020073, accessed on 8 July 2024). Before any procedure, patients were required to read, understand and sign an informed consent form. The study was carried out according to the Helsinki Declaration of 1964 and its subsequent amendments.

**Figure 1:**
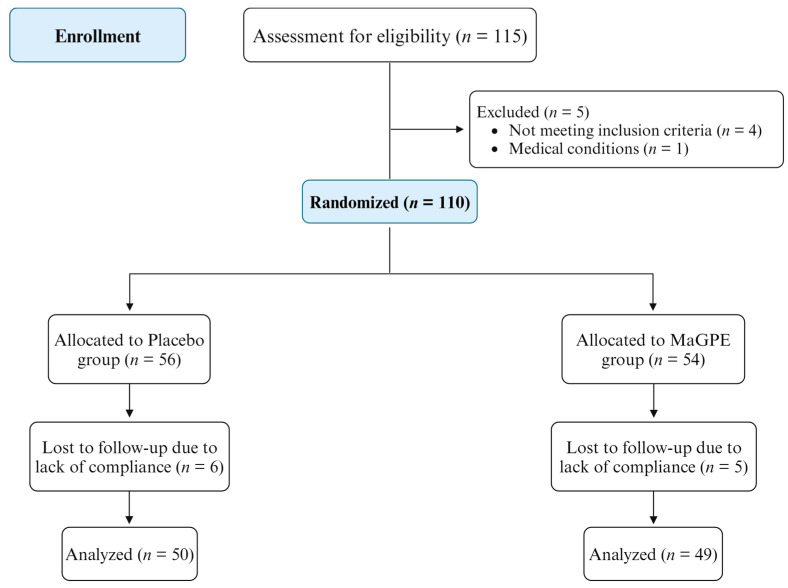
Study’s Consolidated Standards of Reporting Trials (CONSORT) flow diagram.

The inclusion criteria for the study were as follows: age over 18 years, a confirmed diagnosis of nonproliferative DR based on the International Clinical Diabetic Retinopathy (ICDR) and Diabetic Macular Edema Severity Scale, with center-involved DME [24] despite treatment with dexamethasone (DEX) injections. Clinically severe macular edema, as defined by the ETDRS criteria, refers to the thickening of the retina that affects the central area of the macula [25]. Exclusion criteria included the presence of other retinal diseases with macular edema; confirmed or suspected ocular or periocular infection; advanced glaucoma; aphakia; eyes with anterior chamber intraocular lenses; scleral-fixated and iris-fixated intraocular lens; rupture of the posterior capsule; recent ocular surgery (within the last 3 months); ischemic maculopathy; severe hepatic, renal and cardiovascular diseases; other chronic degenerative diseases such as cancer; pregnancy; suspicion of pregnancy; breastfeeding; birch pollen allergy; and the use of medications or supplements containing grape polyphenols. Patients were advised to maintain their usual diet and lifestyle throughout the study period. Clinical assessments, including general medical evaluations, ophthalmic examinations, anthropometric measurements and blood sampling, were performed at the beginning of the study (T0) and after 6 months of MaGPE treatment (T6). To ensure a blinded data analysis, the researchers conducting the blood tests and the physicians performing the clinical assessments were unaware of the patients’ group assignments. Additionally, a separate researcher carried out the statistical data analysis. To determine the simple size of the study, a priori power analysis was performed based on the data of the study of Carnevali et al. [26]. As shown in Figure 1, a total of 115 patients were initially screened for inclusion in the study. However, 6 of these patients were deemed ineligible (HbA1c > 10%). Consequently, 110 patients received the assigned intervention, and 99 successfully completed the study (11 patients lost to follow-up). Simple randomization was carried out with an enrollment ratio of 1:1.

### 2.2. Formulation of MaGPE Supplement

The MaGPE formulation consisted of MaGPE, which is patented (n°102020000006493). Specifically, MaGPE was derived from Aglianico cultivar grapes harvested and collected in autumn of 2020. The large-scale production was carried out by MBMed Company (Turin, Italy). For the polyphenol extract production, grapes were extracted with water at 50 °C, followed by filtration, concentration and a spray-drying process with maltodextrins as support (40–70%) to obtain a fine microencapsulated powder. As previously reported [27], the High-Performance Liquid Chromatography–Diode Array Detector (HPLC-DAD, Jasco Inc., Easton, MD, USA) analysis of the polyphenol profile of MaGPE revealed the presence of the following polyphenols: procyanidin B2 400.1 ± 40.2 µg/g, catechin 2546.2 ± 301.5 µg/g, epicatechin 1811.5 ± 197.1 µg/g, resveratrol 8.5 ± 1.2 µg/g, quercetin 193.2 ± 1.97 µg/g, quercetin-3-*O*-glucoside 215.3 ± 19.5 µg/g, procyanidin B1 1116.9 ± 83.3 µg/g, procyanidin C1 1477.1 ± 179.0 µg/g, *p*-coumaric acid 274.6 ± 29.0 µg/g, syringic acid 1062.5 ± 82.0 µg/g, gallic acid 714.1 ± 38.8 µg/g, caffeic acid 246.8 ± 10.4 µg/g. Patients who satisfied the inclusion criteria were enrolled and divided into 2 groups: the first group received two gastric-resistant oral tablets containing 400 mg of MaGPE twice daily (MaGPE group), while the second group assumed the same amount of maltodextrins (Placebo group). Patients of both groups were asked to maintain the regimen of intravitreal injection.

### 2.3. Assessment of Ophthalmic Outcomes

The study evaluated anatomical and functional changes, specifically BCVA, CRT and vascular perfusion (VP), at baseline and during the follow-up timepoints. These changes were then compared between the 2 groups. Specifically, all patients underwent a comprehensive examination, including BCVA assessment using a 3 m logarithmic visual acuity chart with an Early Treatment Diabetic Retinopathy System (ETDRS) chart, slit lamp examination, intraocular pressure measurement, and fundus evaluation through indirect ophthalmoscopy. The RTVue OCT (Optovue Inc., Fremont, CA, USA) was utilized to perform Optical Coherence Tomography (OCT). The Optovue algorithm was used to evaluate the values of CRT in retina map swabs. An expert ophthalmologist (A.C.) examined the measurements to verify accurate segmentation. OCT–Angiography (OCTA) was performed using the XR Avanti AngioVue OCT-A (Optovue, Fremont, CA, USA). The OCTA imaging provided quantitative data in the form of macular scans of 3 mm × 3 mm, with the center of the scan focused on the fovea. The software of the instrument (version 2017.1.0.151) automatically divided OCTA scans into en-face slabs. The quantitative vascular measurement of superficial capillary plexus (SCP) consisted of vascular perfusion (VP) (% of area occupied by vessels) in the whole zone for the OCTA 3 mm × 3 mm scan. The quantitative analysis of SCP was conducted using the default settings of the automated software algorithm of the AngioPlex (version 2017.1.0.151).

### 2.4. Measurements of Biochemical and Oxidative Stress Biomarkers

All participants underwent a standardized physical examination, an assessment of their medical history, laboratory tests, and measurements of blood pressure and heart rate. Participants were instructed to record their food supplement intake in a monitoring table for the intervention study and to note any side effects in daily reports. They were advised to abstain from alcohol and vigorous physical activity for 48 h before blood sampling. Participants maintained their usual diet and lifestyle throughout the study. All blood samples were collected in the morning, immediately following heart rate and blood pressure measurements. Blood was drawn from each participant using 3 mL EDTA-coated tubes (Becton–Dickinson, Plymouth, UK). Plasma was immediately separated by centrifugation (20 min, 2200× *g*, 4 °C). Glycated hemoglobin (HbA1c), plasma total cholesterol (TC), low-density lipoprotein cholesterol (LDL-C), high-density lipoprotein cholesterol (HDL-C), triglyceride (TG), alanine aminotransferase (ALT), aspartate aminotransferase (AST), uric acid and creatine levels were determined using commercially available kits from Bionova s.r.l. (Avellino, Italy). Serum levels of reactive oxygen metabolite derivatives (d-ROMs) and oxidized low-density lipoproteins (oxLDLs) were monitored as biomarkers related to oxidative stress. Analyses for both d-ROMs and oxLDLs were performed using an automated analyzer (Free Carpe Diem, Diacron International, Grosseto, Italy) with commercially available kits (Diacron International), following the manufacturer’s protocols as previously described [28]. To conduct the d-ROM test, 10 µL of serum was added to 1 cm cuvettes containing 1 mL of R2 reagent (acetate buffer, pH 4.8). The mixture was gently mixed, and 20 µL of R1 reagent (a chromogenic mixture consisting of aromatic alkyl-amine, A-NH_2_) was added. The cuvettes were inverted to mix the contents, and the samples were then read at 546 nm (5 min, 37 °C) using an automated analyzer. For the LP-CHOLOX test, 10 µL of serum was added to a plastic tube containing 1 mL of R1 reagent (indicators mixture) and two drops of R2 reagent (reduced iron). The mixture was shaken to mix, incubated at 37 °C for 2 min and centrifuged at 1400× *g* for 2 min. The supernatants were then transferred into 1 cm cuvettes and read at 505 nm (37 °C) using an automated analyzer. A blank was prepared using the same procedure without the addition of the sample.

### 2.5. Statistical Analysis

Statistical analysis was performed using GraphPad Prism version 8.4.3. (GraphPad Software Inc., San Diego, CA, USA). Unless otherwise stated, all the experimental results were expressed as the mean ± standard deviation (SD). The Anderson–Darling and Kolmogorov–Smirnov tests were applied to assess whether data were normally distributed. Statistical analysis of the data was performed by Student’s *t*-test. A two-way ANOVA test followed by a Tukey–Kramer and Bonferroni post hoc test was used to analyze changes in ophthalmic measurements at the different time points. Significance was accepted at the 5% level.

## 3. Results

### 3.1. Evaluation of Clinical and Biochemical Parameters in Placebo and MaGPE Groups

Table 1 summarizes the clinical and biochemical parameters of participants in both the Placebo (*n* = 50) and the MaGPE groups (*n* = 49) at baseline (T0) and after 6 months (T6). At baseline, the 2 groups showed no significant differences in any of the measured parameters. The Placebo group had a mean age of 66.4 ± 8.1 years, while the MaGPE group had a mean age of 67.5 ± 8.7 years. The HbA1c levels were 7.9 ± 1.5% for the Placebo group and 6.8 ± 0.4% for the MaGPE group. Other parameters, such as HDL-C, LDL-C, TG, TC, ALT, AST and creatinine, also showed comparable values between the groups, indicating that the groups were well-matched at the beginning of the study. The biochemical parameters remained relatively stable in both groups, with no significant variations observed at T6 compared to baseline.

### 3.2. Changes in Ophthalmic Parameters in Placebo and MaGPE Groups

**Figure 2:**
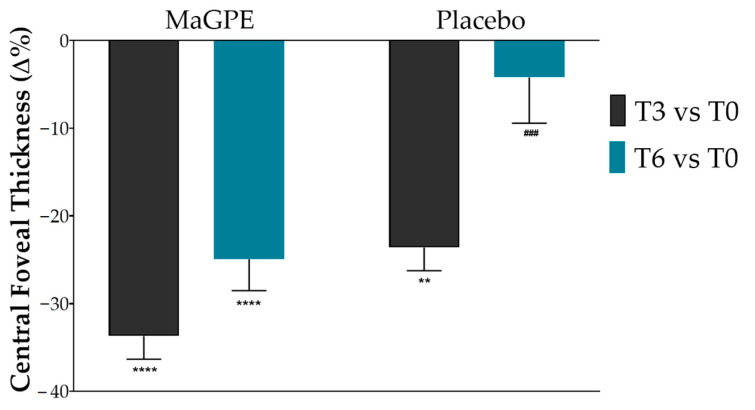
Percentage of changes in central foveal thickness after 3-month (T3) and 6-month (T6) treatment in the group treated with DEX injection + MaGPE nutraceutical formulation (MaGPE group) and the group treated with DEX injection + maltodextrins (Placebo group). Data are expressed as mean ± SEM. Statistical significance was calculated by 2way ANOVA followed by Tukey’s multiple comparisons test. ** *p* ≤ 0.01, **** *p* < 0.0001, significantly different vs. T0 within each group; ### *p* ≤ 0.001, significantly different vs. MaGPE group at the same time point of analysis.

The effectiveness of the administered treatment was evaluated by comparing changes in CRT and BCVA values between the MaGPE treatment group and the Placebo group. As reported in Figure 2, the MaGPE group showed a notable reduction in CRT across T3 and T6 time points. In particular, at T3, patients showed a signification reduction in CRT values of approximately 33.7% (from 492.6 μm, 95% CI 463.4–521.8, to 326.8 μm, 95% CI 298.3–355.3, *p* < 0.0001 vs. baseline) and 24.6% at T6 (from 492.6 μm, 95% CI 463.4–521.8, to 369.8 μm, 95% CI 332.6–406.9, *p* < 0.0001 vs. baseline).

**Figure 3:**
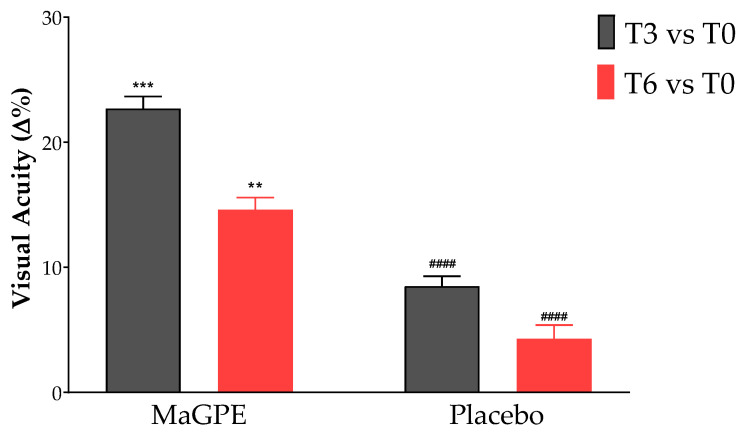
Percentage of changes in visual acuity after 3-month (T3) and 6-month (T6) treatment in the group treated with DEX injection + MaGPE nutraceutical formulation (MaGPE group) and the group treated with DEX injection + maltodextrins (Placebo group). Data are expressed as mean ± SEM. Statistical significance was calculated by 2way ANOVA followed by Tukey’s multiple comparisons test. ** *p* < 0.01, *** *p* < 0.001, significantly different vs. T0 within each group; #### *p* ≤ 0.0001, significantly different vs. MaGPE group at the same time point of analysis.

Additionally, the BCVA also showed improvements (Figure 3). Specifically, BCVA values improved by 23.0% at T3 (from 0.274, 95% CI 0.222–0.329 to 0.337, CI 0.263–0.392 degrees, *p* < 0.001 vs. baseline), although there was a slight decrease to 14.6% at T6 (from 0.274, 95% CI 0.222–0.329 to 0.314, 95% CI 0.252–0.359 degrees, *p* < 0.01 vs. baseline). In the Placebo group, the CRT reduction percentages were lower, with a 23.6% reduction in CRT at T3 (from 395.0 μm, 95% CI 358.1–432.8, to 302.1 μm, 95% CI 281.0–323.2, *p* < 0.01 vs. baseline) and a reduction of 4.2% at T6 (from 395.0 μm, 95% CI 358.1–432.8, to 379.4 μm, 95% CI 337.5–420.4, ns vs. baseline). Similarly, the BCVA changes in the control group were less pronounced compared to the MaGPE group, with an 8.5% and 4.3% improvement, respectively, at the T3 and T6 time points (from 0.287, 95% CI 0.230–0.342 to 0.311, 95% CI 0.262–0.372 for T3 and from 0.287, 95% CI 0.230–0.342 to 0.299, 95% CI 0.242–0.390 for T6, ns vs. baseline). Moreover, statistically significant differences were observed between the two groups as regards the ∆% of CRT values at T6, +24.6% vs. 4.2% (*p* < 0.001) and BCVA values both at T3, 23.0% vs. 8.5%, (*p* < 0.0001) and T6, 14.6% vs. 4.3% (*p* < 0.0001) for MaGPE and Placebo groups, respectively.

In addition, Table 2 shows data on all ophthalmic parameters evaluated in the study at time points T0, T3 and T6. Beyond the previously reported changes in CRT and BCVA, notable modifications were also observed in the percentage of VP over time. Specifically, the Placebo group exhibited a decreasing trend in vascular perfusion, −4.4% and −5.8% at T3 and T6, respectively, compared to baseline. In contrast, the MaGPE group demonstrated stabilization in VP percentage, with slight increases at T3 and T6, +3.0% and +2.7% at T3 and T6, respectively, compared to baseline.

### 3.3. Oxidative Stress Biomarker Modulation by MaGPE Supplementation

In addition to the primary ocular outcomes, an evaluation of oxidative stress biomarkers was conducted to further understand the systemic effects of MaGPE supplementation. Specifically, the circulating dROM and ox-LDL levels were assessed at different timepoints. As presented in Table 3, the MaGPE group exhibited a significant reduction in both dROM and oxLDL levels over the study period. The dROM levels decreased from 1100.6 ± 430.1 UCARR at T0 to 974.8 ± 390.2 UCARR at T3 and further to 930.6 ± 310.3 UCARR at T6 (*p* < 0.05 vs. T0). Similarly, oxLDL levels in the MaGPE group decreased from 953.9 ± 212.4 µEq/L at T0 to 867.0 ± 209.5 µEq/L at T3 and markedly to 735.0 ± 213.7 µEq/L at T6 (*p* < 0.0001 vs. T0). In contrast, the Placebo group did not exhibit significant changes in these biomarkers. The dROM levels remained relatively stable, with 1112.5 ± 450.2 UCARR at T0, 1095.0 ± 465.4 UCARR at T3, and 1115.0 ± 464.3 UCARR at T6. Similarly, oxLDL levels in the Placebo group showed negligible variations, with 974.8 ± 208.3 µEq/L at T0, 962.0 ± 213.4 µEq/L at T3, and 978.0 ± 201.5 µEq/L at T6.

## 4. Discussion

DR represents a significant complication of diabetes mellitus, impacting a substantial portion of the diabetic population and representing a notable public health concern [29]. Projections indicate an escalating trend in DR prevalence, in concomitance with the predicted growth of the global diabetic population [3,30]. This trend underscores the urgent need for integrated DR management strategies to address this public health challenge and reduce preventable blindness in the diabetic population. Despite advancements in therapeutic modalities, DR remains a significant healthcare challenge, exerting considerable strain on healthcare resources and affecting patient quality of life. While existing treatments offer benefits in certain scenarios, they are not free from limitations, including the need for repeated interventions and potential adverse effects [31]. Moreover, the limited effectiveness of current pharmacological treatments for DR in addressing oxidative stress underscores the critical need for alternative therapeutic approaches.

The findings from this study indicate a pronounced beneficial effect of MaGPE supplementation on patients with DR, particularly in terms of reducing CRT and improving BCVA values. The MaGPE group exhibited substantial reductions in CRT at both T3 and T6, with reductions of 33.7% and 24.6%, respectively, compared to baseline values (*p* < 0.0001). These significant reductions suggest that MaGPE effectively decreases retinal swelling, which is a critical factor in managing DR. Notably, the Placebo group showed a 23.6% reduction at T3 but only a 4.2% reduction at T6, indicating limited long-term efficacy without active treatment. Additionally, the BCVA improvements further support the efficacy of MaGPE. At T3, the MaGPE group showed a 23.0% improvement in BCVA (*p* < 0.0001), which, although reduced to 14.6% at T6, still indicated overall better outcomes compared to the Placebo group. The Placebo group’s BCVA improvements were 8.5% at T3 and 4.3% at T6, demonstrating less pronounced enhancements in visual acuity. The statistically significant differences observed between the two groups of the study suggest that the MaGPE supplementation not only provides immediate benefits but also sustains its effectiveness over a more extended period, which is crucial for long-term management of DR. In addition to the previously reported changes in CRT and BCVA, further modifications were observed in the percentage of vascular perfusion over time, as detailed in Table 2. The Placebo group exhibited a decreasing trend in VP over the study period. In contrast, the MaGPE group demonstrated stabilization of the VP percentage, with slight increases observed at both T3 and T6, compared to baseline. Although the increases observed in the MaGPE group were not statistically significant, this upward trend is a promising indicator of potential improvement in DR. The stabilization and slight enhancement of VP suggest that MaGPE supplementation may help maintain vascular integrity in patients with DR. These results align with previous studies suggesting that polyphenolic compounds, such as those found in grape pomace, exhibit antioxidative and anti-inflammatory properties that are beneficial in reducing retinal oxidative stress and improving vascular integrity [23,32].

In this regard, oxidative stress results from an imbalance between the production of reactive oxygen species (ROS) and the body’s antioxidant defenses, leading to cellular damage and dysfunction within the retina [33]. By targeting oxidative stress, these alternative approaches have the potential to arrest or slow down the progression of retinal damage, ultimately preserving vision and improving patient outcomes [15]. Furthermore, addressing oxidative stress may have broader implications beyond DR, as it is also implicated in the pathogenesis of other diabetic complications, such as nephropathy and neuropathy [34]. Currently, pharmacological treatments for the management of DR do not address the underlying oxidative stress condition, which is a pivotal factor in the disease’s pathogenesis. In contrast, these results demonstrated the efficacy of MaGPE in mitigating oxidative stress. Specifically, significant modulation of oxidative stress biomarkers has been reported in patients supplemented with the nutraceutical formulation. The MaGPE group exhibited a substantial reduction in both biomarkers over the study period (d-ROMs and oxLDL levels). In contrast, the Placebo group did not show significant changes in these biomarkers, with d-ROMs and oxLDL levels remaining relatively stable throughout the study period. These findings suggest that MaGPE supplementation effectively reduces oxidative stress in DR patients, which could contribute to its overall therapeutic efficacy in improving retinal health and visual outcomes [15,16,18]. Therefore, given the complex pathophysiology of diabetic retinopathy, which involves multiple mechanisms including oxidative stress and VEGF-mediated pathways [4], the multifaceted action of MaGPE—relevant in the inhibition of a-glucosidase activity—as a potent antioxidant and inhibitor of carbohydrate-metabolizing enzymes, could address several aspects of disease progression [35].

In line with our observations, a previous study conducted by our group assessed the therapeutic benefits of oral supplementation with bromelain and curcugreen as sources of antioxidant compounds in patients with non-proliferative DR exhibiting focal DME [26]. The randomized cohort consisted of 33 patients divided into two groups, one receiving the oral supplement and the other serving as a control group under standard observation. Over a 12-month period, key outcomes such as BCVA and CRT assessed via OCT and vascular perfusion in both the superficial and deep capillary plexuses (SCP and DCP, respectively), measured by OCT–Angiography (OCTA), were analyzed. The results revealed a statistically significant interaction between the duration of the study and the treatment on CRT and DCP values, revealing marked improvements in these parameters within the treatment group over time. Nevertheless, no significant changes were observed in BCVA and SCP. Other polyphenolic compounds have demonstrated potential in mitigating oxidative stress associated with DR [15]. As an example, quercetin, a flavonoid abundant in various plant-based foods, has shown promise in preventing DR by enhancing antioxidant enzyme expression, inhibiting NF-κB and Caspase-3 activation, and protecting against diabetes-induced retinal neurodegeneration and oxidative damage [36]. Similarly, resveratrol, a nonflavonoid polyphenol abundant in grapes, has been investigated for its protective effects against age-related ocular diseases, including DR [37,38]. Specifically, an in vitro study performed by Li et al. highlighted the ability of resveratrol to inhibit endoplasmic reticulum stress, which plays a crucial role in retinal vascular degeneration [39]. Additionally, Losso and colleagues investigated the anti-inflammatory effects of resveratrol on RPE cells under hyperglycemic conditions. Their study revealed that resveratrol significantly reduced the levels of VEGF, TGF-β1, COX-2, IL-6 and IL-8 in a dose-dependent manner. Furthermore, resveratrol inhibited the activity of protein kinase *C*-beta (PKCβ), known to increase VEGF activity under hypoxic conditions, thereby helping to preserve the integrity of the blood–retina barrier [40]. Overall, these findings further support the potential of the MaGPE nutraceutical formulation, which is rich in polyphenolic compounds (e.g., resveratrol and quercetin), as a therapeutic agent for the management of DR.

This is a randomized, placebo-controlled clinical trial, which is crucial for assessing the quality of the study. However, it is important to note that our study is limited by its monocentric nature. The presence of a single center may restrict the applicability of the findings to different populations.

## 5. Conclusions

An increasing body of evidence underscores both research and clinical interest in identifying nutraceutical approaches for managing DR. The investigation of nutraceuticals, particularly those derived from grape pomace, offers promising avenues to enhance clinical management strategies for this sight-threatening condition. The significant improvements observed for CRT and BCVA values in the MaGPE group, compared to the Placebo group, demonstrate its potential as an effective intervention for reducing retinal swelling and enhancing visual acuity in patients with DR. Therefore, these findings support the potential of MaGPE supplementation as a protective strategy to mitigate the progression of DR in association with standard DR therapies, particularly through the enhancement of the anatomical and functional integrity of the retina. Further studies could be conducted to gain a better understanding of the molecular and cellular mechanisms underlying the observed effects.

## 6. Patents

Patent n°102020000006493. Patent Title: “Food supplement to counteract age-related macular degeneration”. Inventors: Ettore Novellino and Gian Carlo Tenore. Publication Date: 21 March 2023.

## Figures and Tables

**Table 1:** Comparison of baseline (T0) and six-month (T6) follow-up parameters between Placebo and MaGPE groups.

Parameters	Placebo Group (*n* = 50)		MaGPE Group (*n* = 49)		
	T0	T6	T0	T6	
Male, n° (%)	34 (68.0)	-	35 (71.4)	-	ns
Age (years)	66.4 ± 8.1	-	67.5 ± 8.7	-	ns
HbA1c (%)	7.9 ± 1.5	7.2 ± 4.1	6.8 ± 0.4	7.3 ± 3.1	ns
HDL-C (mg/dL)	36.3 ± 10.4	35.4 ± 11.2	37.0 ± 14.2	35.9 ± 9.6	ns
LDL-C (mg/dL)	98.3 ± 38.7	94.1 ± 33.1	108.9 ± 42.7	91.5 ± 32.1	ns
TG (mg/dL)	148.7 ± 64.5	158.5 ± 77.4	157.5 ± 63.6	153.5 ± 85.2	ns
TC (mg/dL)	158.5 ± 34.8	164.7± 29.4	177.4 ± 44.2	158.1 ± 27.7	ns
ALT (UI/L)	18.8 ± 7.6	17.4 ± 9.4	16.7 ± 10.6	17.3 ± 10.8	ns
AST (UI/L)	28.6 ± 13.8	30.0 ± 15.2	29.6 ± 17.8	31.0 ± 16.4	ns
Uric acid (mg/dL)	8.6 ± 6.2	8.4 ± 6.7	8.8 ± 7.2	8.2 ± 6.2	ns
Cre (mg/dL)	1.0 ± 0.5	1.1 ± 0.4	1.2 ± 0.5	1.3 ± 0.6	ns

Data are expressed as mean ± standard deviation. ns, non significant, T6 vs. T0 within the same group. Abbreviations: AST: aspartate aminotransferase; ALT: alanine aminotransferase; Cre: creatinine; HbA1c: glycated hemoglobin; HDL-C: high-density lipoprotein cholesterol; LDL-C: low-density lipoprotein cholesterol; TC: total cholesterol; TGs: triglycerides.

**Table 2:** Evaluation of ophthalmic parameters in Placebo and MaGPE groups at baseline (T0), and after three-month (T3) and six-month (T6) follow-up.

Parameters	Placebo Group (*n* = 50)			MaGPE Group (*n* = 49)		
	T0	T3	T6	T0	T3	T6
CRT (%)	395.0 ± 130.2	302.1 ± 73.4 **	379.4 ± 144.4	492.6 ± 107.1	326.8 ± 99.1 ****	369.8 ± 134.7 ****
BCVA (letters)	0.287 ± 0.19	0.311 ± 0.19	0.299± 0.26	0.274 ± 0.20	0.337 ± 0.23 ***	0.314 ± 0.20 **
VP (%)	38.9 ± 5.9	37.2 ± 7.0	36.7 ± 6.8	37.9 ± 5.8	39.0 ± 5.7	38.9 ± 7.5

Data are expressed as mean ± standard deviation. ** *p* < 0.01, *** *p* < 0.001, **** *p* < 0.0001 significantly different vs. T0 within the same group. Abbreviations: BCVA, best-corrected visual acuity; CRT, central retinal thickness; VP, vascular perfusion.

**Table 3:** Assessment of biomarkers of oxidative stress in Placebo and MaGPE groups at baseline (T0), and after three-month (T3) and six-month (T6) follow-up.

Parameters	Placebo Group (*n* = 50)			MaGPE Group (*n* = 49)		
	T0	T3	T6	T0	T3	T6
dROMs (UCARR)	1112.5 ± 450.2	1095.0 ± 465.4	1115.0 ± 464.3	1100.6 ± 430.1	974.8 ± 390.2	930.6 ± 310.3 *
ox-LDL (µEq/L)	974.8 ± 208.3	962. ± 213.4	978.0 ± 201.5	953.9 ± 212.4	867. ± 209.5 *	735.0 ± 213.7 ****

Data are expressed as mean ± standard deviation. * *p* < 0.05, **** *p* < 0.0001 significantly different vs. T0 within the same group. Abbreviations: dROMs, reactive oxygen metabolite derivatives (DROMs), and oxidized low-density lipoprotein (ox-LDL).

## Data Availability

The data presented in this study are available on request from the corresponding author. The data are not publicly available due to privacy restrictions.
